# Laterality of injectate spread in bilateral ultrasound-guided thoracic paravertebral block: a pilot cadaveric study

**DOI:** 10.1186/s40981-026-00849-5

**Published:** 2026-02-05

**Authors:** Asako Nitta, Atsushi Sawada, Sho Kumita, Yuki Ohsaki, Michiaki Yamakage

**Affiliations:** 1 Department of Anesthesiology, Sapporo Medical University School of Medicine, West 16, South 1, Chuo-Ku, Sapporo, Japan; 2 Division of Anesthesia, Gorinbashi Orthopedic Hospital, 2-1, Kawazoe, Minami-Ku, Sapporo, Japan; 3 Division of Cell and Tissue Morphology, Department of Anatomy, Sapporo Medical University School of Medicine, West 17, South 1, Chuo-Ku, Sapporo, Japan

**Keywords:** Thoracic paravertebral block, Thoracic paravertebral space, Ultrasound, Anatomy, Cadaver

## Abstract

**Background:**

Although thoracic paravertebral block (TPVB) is an established regional analgesia technique in thoracic surgery, the extent of injectate spread is variable. Furthermore, while it is known that a larger capacity exists in the left thoracic paravertebral space (TPVS) compared with the right due to mediastinal asymmetry, it remains unclear whether this characteristic translates to functional differences in injectate spread.

**Methods:**

We performed bilateral TPVB in twelve Thiel-embalmed cadavers using an intercostal approach—six were placed in the left lateral decubitus position and six in the right lateral decubitus position. To control for positional bias, 20 mL of dye was administered to both the upper and lower sides while maintaining the same position. The numbers of stained vertebral segmental TPVSs and intercostal spaces (ICSs) were compared between the left and right sides.

**Results:**

TPVB performed on the left side demonstrated significantly more stained segments (median [interquartile range]) than that performed on the right side (TPVS: 4.0 [3.0–5.0] vs. 2.0 [1.0–3.0], *p* = 0.01; ICS: 4.0 [3.0–5.0] vs. 3.0 [2.0–4.0], *p* = 0.04). This tendency remained consistent regardless of the injection side (upper or lower). In addition, we consistently observed extensive dye distribution when the parietal pleura medial to the transverse process was markedly displaced, irrespective of laterality.

**Conclusion:**

This study demonstrates a significant laterality in injectate spread during TPVB, with more extensive longitudinal distribution on the left side. Our findings may provide important insights for understanding the variability of injectate distribution in TPVB.

## Introduction

The thoracic paravertebral block (TPVB) provides unilateral thoracic analgesia through the administration of local anesthetics to the thoracic paravertebral space (TPVS), which is located on either side of the vertebral column. The space is wedge-shaped, longitudinal, contiguous, and bordered by the superior costotransverse ligament, parietal pleura, ribs, and the vertebral column. The TPVS contains intercostal nerves, sympathetic chain, and dorsal rami of the spinal nerves [1, 2]. TPVB has long relied on the landmark technique, which entails potential risks such as block failure, inadvertent pleural puncture, and pneumothorax [3]. Over the past two decades, ultrasound-guided TPVB has gained widespread acceptance because of its procedural safety, clear visualization of the parietal pleura and TPVS through ultrasound imaging, and higher success rate than the landmark technique [4]. Furthermore, TPVB has now become preferred over epidural block as a perioperative analgesic method in thoracic surgery because of the lower risk of complications, such as hypotension, urinary retention, and nausea or vomiting, along with its adequate analgesic efficacy [5, 6].

However, TPVB is associated with significant variability in the extent of injectate spread [7–9]. Previous studies have compared among different ultrasound-guided TPVB approaches: parasagittal [10], intercostal [11], and paralaminar [12]. One study reported that it is comparable among the three approaches [9], whereas others have found that the paralaminar approach produces a more extensive spread than the intercostal approach [13].

Aside from technical variations, the factors contributing to injectate diffusion in TPVB have not been sufficiently investigated. One potential factor is the laterality of the TPVS dimensions. An anatomical study using computed tomography have demonstrated that the left TPVS is wider than the right, as the descending aorta shares connective tissue with the left TPVS [14]. However, it remains unclear whether this morphological asymmetry translates to functional differences in injectate spread during TPVB.

To address this question, we conducted a pilot cadaveric study to investigate how the asymmetry of the TPVS influences injectate distribution during bilateral TPVB performed in the lateral position. To accurately assess laterality, it is essential to minimize procedural variations associated with patient positioning. Accordingly, we employed an experimental design in which bilateral TPVBs were performed while maintaining the same lateral decubitus position.

## Materials and methods

### Ethical considerations

This cadaveric study was approved by the Ethics Committee of Sapporo Medical University on January 25, 2022, August 22, 2022, and August 19, 2024 (Approval Nos. 3–1–61, 4–1–49, and 6–1–39). Written informed consent was obtained from the family of each cadaver donated to the body donation program (Shiragiku-kai) run by Sapporo Medical University. Twelve Thiel-embalmed adult cadavers (four males and eight females) without visible back scars or a history of thoracic or spine surgery were included in the study.

## Anatomical dissection

All cadavers were dissected in the supine position before injection so that the thoracic cavities could be observed simultaneously immediately after injection. A coronal skin incision was made along both thoracic cages at the level of the posterior axillary line, and both caudal edges of the incision were extended medially and connected. The muscles comprising the anterior thoracic and abdominal walls were dissected from both costal margins. Next, we dissected the ribs from the second to twelfth vertebrae along the incision line using a costotome to create an anterior costal flap. The flap was elevated, and the trachea, esophagus, common carotid arteries, and jugular veins were disconnected at the level of the hyoid bone. The organs within the thoracic cage (lungs, heart, trachea, esophagus, aorta, and large vessels) were carefully dissected caudally to prevent injury to the posterior parietal pleura and were subsequently returned to their original positions.

### Ultrasound-guided bilateral TPVB procedure

**Fig. 1:**
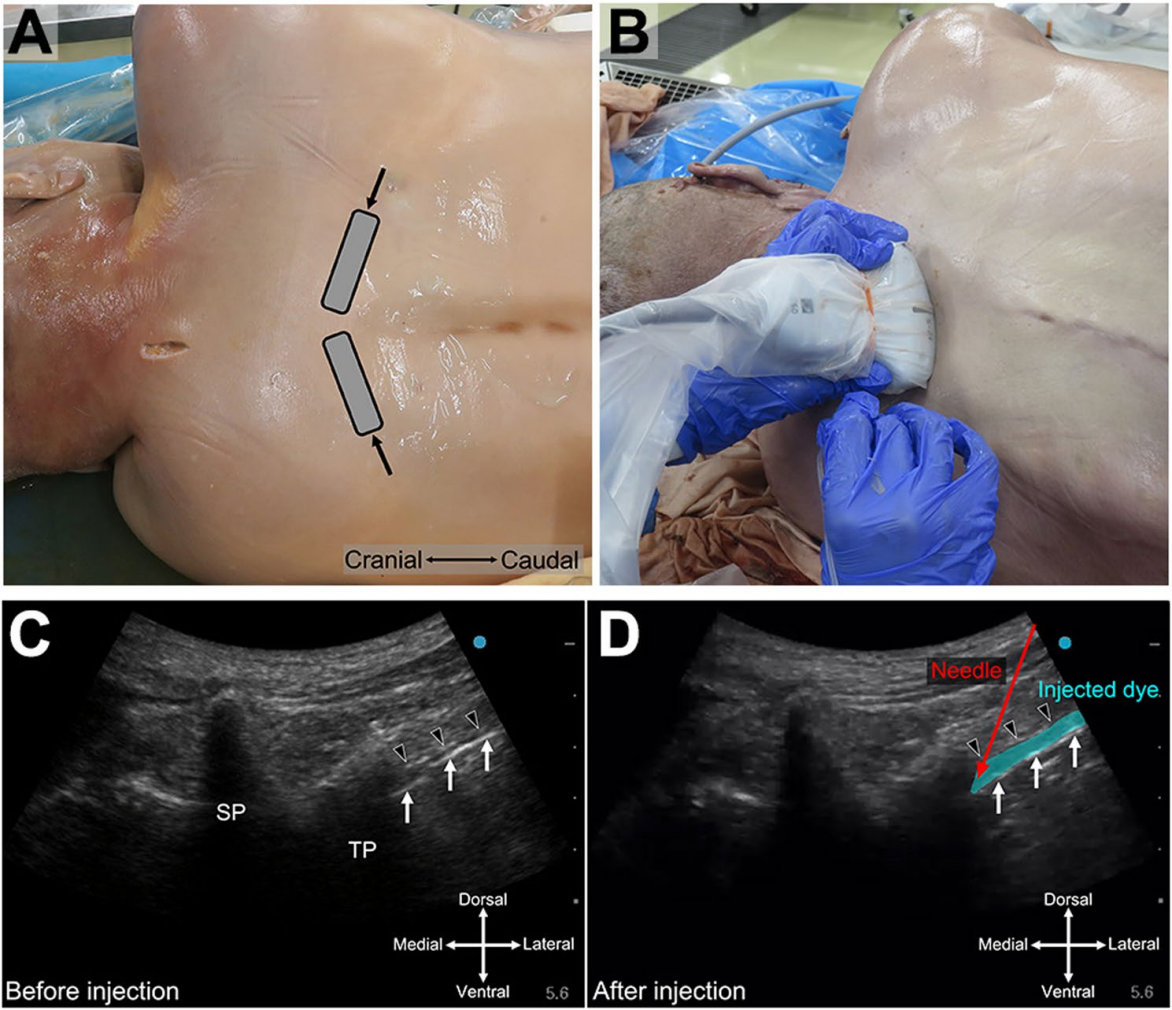
Photographs demonstrating bilateral TPVB in the lateral position and ultrasound images of the TPVB using an intercostal approach. **A** A low–frequency convex transducer (gray rectangle) was positioned along the intercostal space. The needle (arrows) was inserted in the lateral-to-medial direction from the lateral edge of the transducer using an in-plane technique. **B** Example of an injection administered from the lower side in the lateral position. **C** and **D** show pre-scanning and post-dye injection images, respectively. White arrows and black arrowheads indicate the parietal pleura and internal intercostal membranes, respectively. SP: spinous process; TP: transverse process

After preparing the cadavers as described above, each was allocated to either the left or right lateral decubitus position. Six of the twelve cadavers were positioned in the left lateral decubitus position, and the remaining six in the right, with the upper arm positioned downward to enlarge the interscapular space (Fig. 1A and B). To investigate the influence of laterality on injectate spread while strictly controlling for the confounding effect of positioning bias, injections were administered to both the upper (non-dependent) and lower (dependent) sides while maintaining the same lateral decubitus position.

All cadavers underwent ultrasound-guided bilateral TPVB using an intercostal approach [11]. First, we identified the target vertebral level by palpating the C7 spinous process and counting caudally. We confirmed it by ultrasound imaging, using a low–frequency convex transducer rC60xi 2–5 MHz (Sonosite SⅡ; Fujifilm SonoSite, Tokyo, Japan); we identified the second rib and then counted caudally to determine if the vertebral level was identical to the palpation method. To visualize the transverse process (TP), parietal pleura, and internal intercostal membrane (IIM), which form the dorsal border of the TPVS and are located lateral to the superior costotransverse ligament, the transducer was positioned parallel to the intercostal space (ICS), slightly lateral to the spinous process. Ultrasonography identified the TPVS as a wedge-shaped hypoechoic space surrounded by TP, IIM, and parietal pleura (Fig. 1C).

All injections were performed by a single anesthesiologist with extensive knowledge and experience in ultrasound-guided TPVB (AN). A 22-gauge 8-cm echogenic needle (Ultraplex 360; B. Braun, Melsungen, Germany) was inserted from the lateral side of the transducer using the in-plane technique. When the needle punctured the IIM, a small volume of normal saline was administered as a test dose. After confirming that the needle tip was in the TPVS by observing that the parietal pleura was pushed down, we administered 20 mL of saline-diluted acrylic dye (Fig. 1D). All cadavers were injected into both the upper and lower sides at T4 or adjacent levels, corresponding to the most clearly visualized vertebral level on the ultrasound image. An orange or blue acrylic dye was administered to the upper side, and a green acrylic dye was administered to the lower side.

### Evaluation of dye-spread

Twenty minutes after the injection, the cadavers were placed in a supine position. The anterior costal flap was elevated, and the organs were shifted caudally within the thoracic cage to expose the posterior parietal pleura. The vertebral segmental numbers of the TPVSs and ICSs were recorded. “Deep staining” was defined as dye spread extending entire vertical extent of the segment (from the inferior border of the cranial rib to the superior border of the caudal rib) whereas “faint staining” was defined as staining confined to only part of the segment. All injections and dissections were performed by an experienced anesthesiologist (AS). Two anesthesiologists (AN and AS) assessed the dye-spread using macroscopic observations and photography.

### Statistical analysis

The evaluation outcomes were as follows: (1) the number of stained segmental TPVSs and ICSs and (2) the number of cases in which the dye diffused to the contralateral side of the vertebral column and TPVS. Cadavers with evident pleural punctures were excluded from the analysis. For outcome (1), the number of stained segments included both deep and faint staining because the diluted acrylic dye that diffused into the segment may not have achieved sufficient macroscopic staining. As this was a pilot study and the number of cadavers available for the study was limited, a sample size calculation was not performed. Statistical analyses were performed using GraphPad Prism 10 (GraphPad Software, Boston, MA, U.S.A.). The Wilcoxon matched-pairs signed-rank test was employed to compare the segmental numbers of stained TPVSs and ICSs between the left side (i.e., the lower side of the left lateral decubitus position and the upper side of the right lateral decubitus position) and the right side (i.e., the upper side of the left lateral decubitus position and the lower side of the right lateral decubitus position) of the cadaver. The same test was also used to compare the upper and lower sides in the lateral decubitus position. Data are presented as medians and interquartile ranges. Fisher’s exact test was employed to compare the proportion of dye spread to the contralateral side between the upper and lower sides. Throughout the study, a two-sided *p*-value of less than 0.05 was considered statistically significant.

## Results

**Fig. 2:**
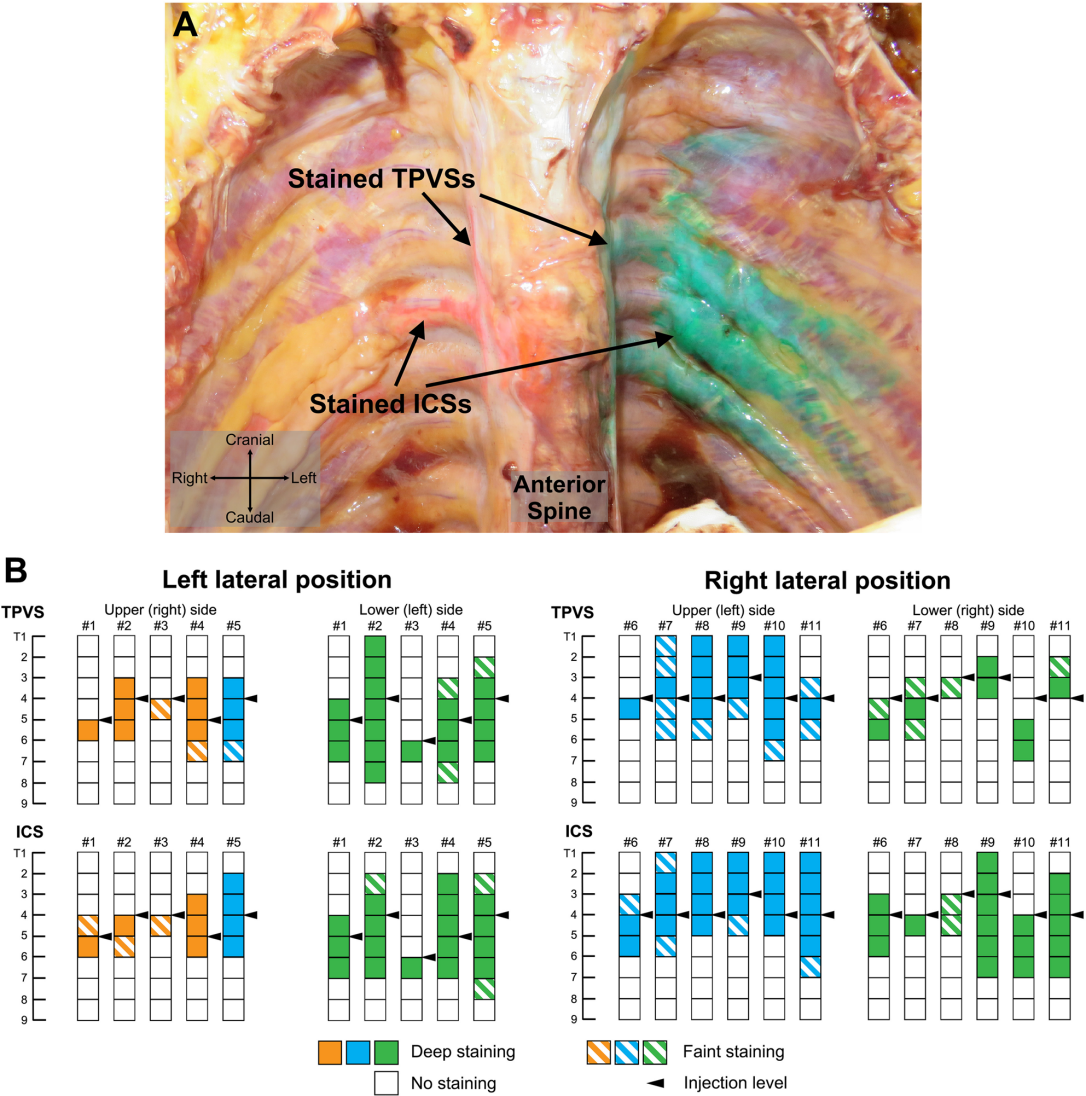
The extent of dye spread following bilateral TPVB. **A** A representative photograph of the anatomical dissection after bilateral TPVB. The orange acrylic dye was administered from the upper-side, and the green was from the lower-side. The organs in the mediastinum (lungs, trachea, heart, aorta, and esophagus) were displaced caudally. **B** Pictorial representation of the range and level of staining dye segments in 11 cadavers. # (Number) indicates the consecutive number of the cadaver. Filled and striped color boxes indicate deep and faint staining, respectively, whereas blanked boxes indicate no staining of the dye macroscopically. Arrowheads indicate the injection site levels. TPVS: thoracic paravertebral space; ICS: intercostal space

Twelve cadavers were examined, including four males and eight females with a mean age of 86.3 years (range, 70–97 years) at the time of death. In eleven of the twelve cadavers, real-time ultrasound imaging confirmed that the injected dye displaced the parietal pleura downward, and subsequent anatomical dissection verified the presence of dye in both the TPVS and ICS (Fig. 2A). One cadaver (female, injected in the left lateral decubitus position) was excluded from the analysis because of a pleural puncture that occurred during the injection from the upper side.

**Fig. 3:**
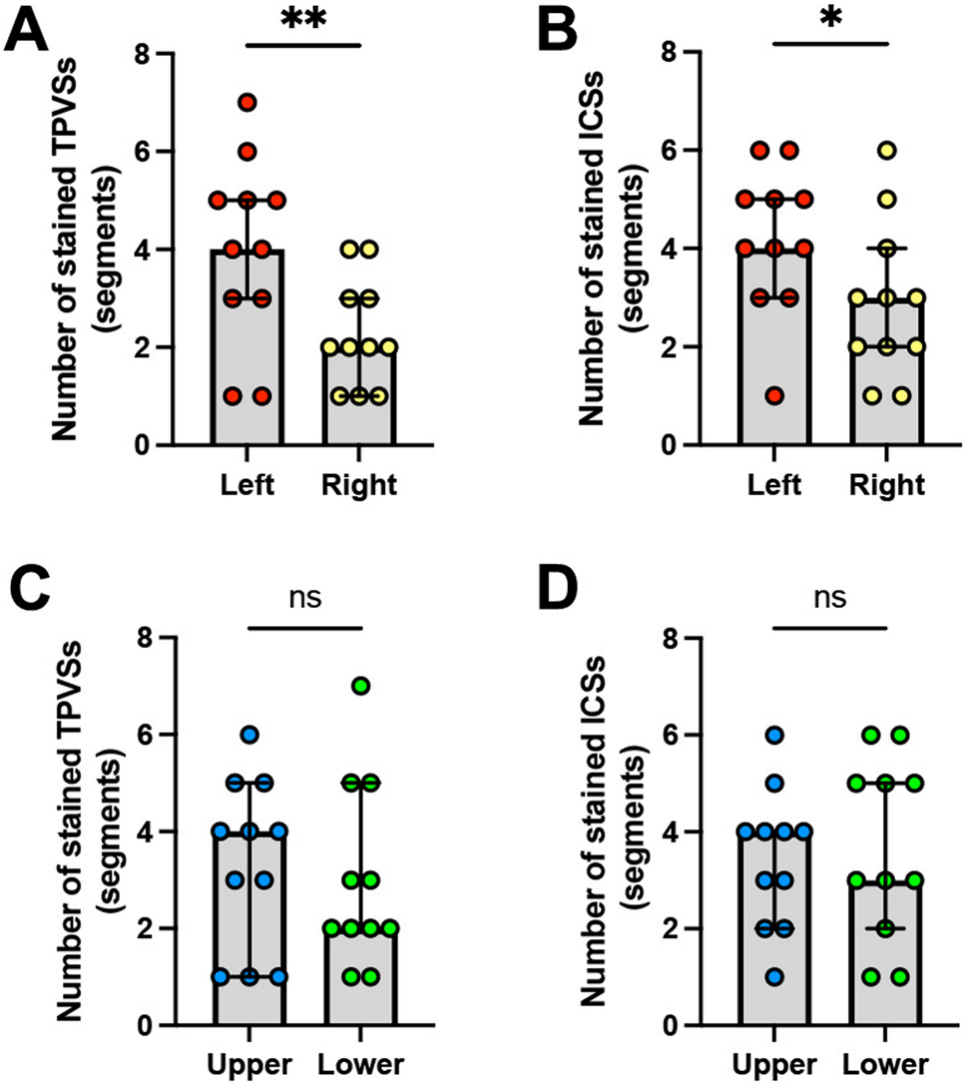
Statistical analysis of the extent of dye diffusion in TPVSs and ICSs. **A** and **B** Comparison of the segmental numbers of stained TPVSs (**A**) and ICSs (**B**) between the left and right sides of the cadaver. *n* = 11 for each group. **C** and **D** Comparison of the segmental numbers of stained TPVSs (**C**) and ICSs (**D**) between the upper and lower sides in the lateral position. Bars and error bars indicate the median values and interquartile ranges, respectively. The colored circles denote the individual data points. *n* = 11 for each group. ns = not significant, p* < 0.05, p** < 0.01 (Wilcoxon matched-pairs signed-rank test). TPVS: thoracic paravertebral space; ICS: intercostal space

Figure 2B illustrates the number of vertebral segments and the levels of dye distribution in the TPVSs and ICSs. A comparison between the left and right sides revealed that the median [interquartile range] number of stained TPVS segments was significantly greater on the left (4.0 [3.0–5.0]) than on the right (2.0 [1.0–3.0]; *p* = 0.01) (Fig. 3A). Similarly, the extent of dye diffusion within the ICSs followed a pattern similar to that observed in the TPVSs, with more extensive diffusion on the left side than on the right (left, 4.0 [3.0–5.0]; right, 3.0 [2.0–4.0]; *p* = 0.04) (Fig. 3B).

To address the potential influence of the injection side (upper vs. lower) on this laterality, we performed a sub-analysis. The results consistently showed wider spread on the left, regardless of positioning. Injections performed on the upper side resulted in a median [interquartile range] of 4.5 [2.5–5.25] stained TPVS segments on the left versus 3.0 [1.0–4.0] on the right, and injections on the lower side resulted in 5.0 [1.0–6.0] segments on the left versus 2.5 [1.75–3.75] on the right.

In contrast to the significant laterality observed, comparison of the upper and lower injection sides in the lateral decubitus position showed no significant difference in the median [interquartile range] number of stained TPVSs (upper, 4.0 [1.0–5.0]; lower, 2.0 [2.0–5.0]; *p* = 0.63) (Fig. 3C). Similarly, there was no significant difference in the median number of stained ICS segments between the upper and lower sides (upper, 4.0 [2.0–4.0]; lower, 3.0 [2.0–5.0]; *p* = 0.69) (Fig. 3D). Moreover, the incidence of stained contralateral vertebral column was not significant (upper, 36.4%; lower, 0%; *p* = 0.09). None of the cases demonstrated the contralateral TPVS stained regardless of the injection side.

**Fig. 4:**
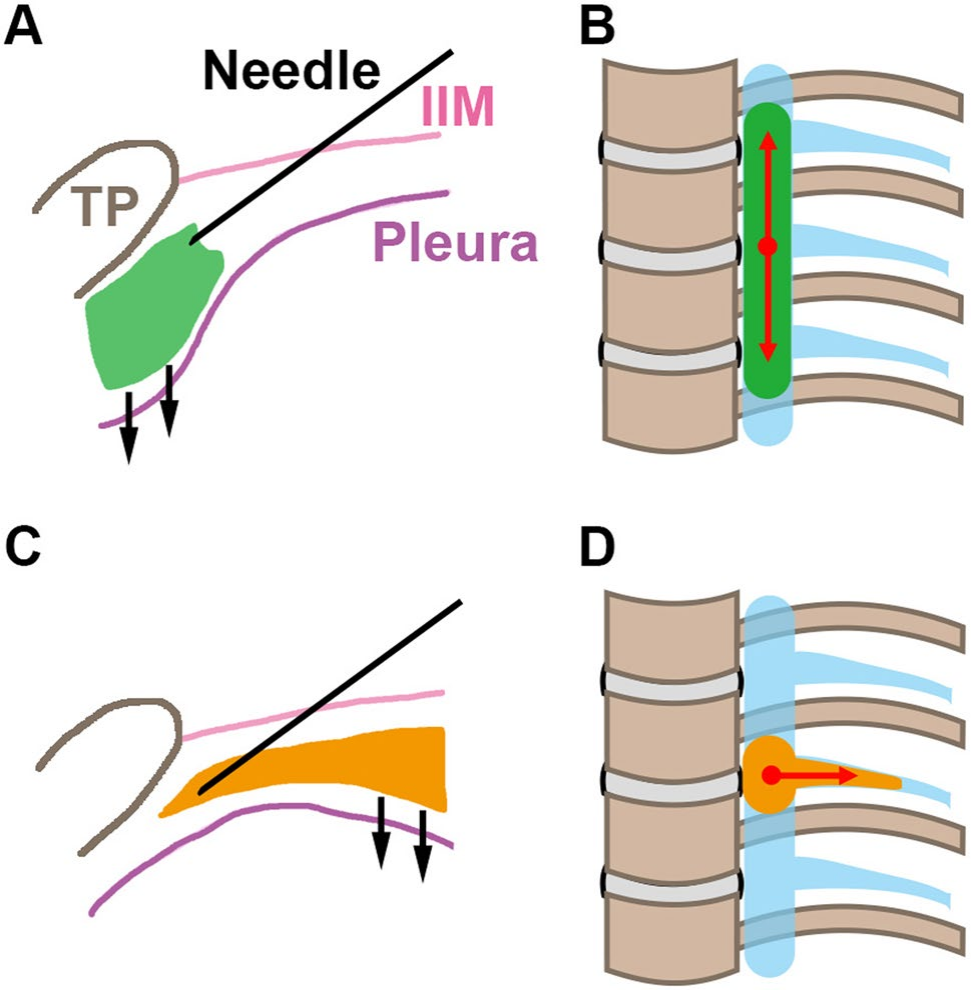
Relationship between pleural displacement and patterns of injectate spread. In the pattern where the pleura beneath and medial to the transverse process was displaced downward (**A**), the injectate showed predominant craniocaudal spread along the TPVS (**B**). By contrast, when the pleura lateral to the transverse process was displaced (**C**), craniocaudal spread along the TPVS was limited, with a tendency for lateral direction into the ICS (**D**). Light blue regions in panels **B** and **D** represent TPVSs. Black arrows in panels **A** and **C** indicate pleural displacement caused by the injectate (shown in green in **A** and **B**; orange in **C** and **D**). Double-headed red arrows in panels **B** and **D** indicate the direction and extent of injectate spread. TP: transverse process; IIM: internal intercostal membrane

Table 1 presents a more detailed analysis of injectate distribution patterns. When real-time ultrasonography confirmed the needle tip immediately above the parietal pleura, the median number of stained TPVS segments was 5.0 on the left and 2.5 on the right. Moreover, when injection preferentially displaced the pleura directly beneath and medial to the TP downward, the injectate spread across multiple vertebral segments in both the TPVS and the ICS (Fig. 4A and B). In contrast, when the pleura lateral to the TP was preferentially displaced, craniocaudal spread along the TPVS was limited (Fig. 4C and D).

**Table 1:** Detailed analysis of the dye-spread pattern

Patterns of injection	Left TPVS / ICS (segment)	Right TPVS / ICS (segment)
Needle tip directly above the pleura	5 / 5 [#4 #7 #8 #10 #11]	2.5 / 3.5 [#5 #8 #10 #11]
Pleura medial to the TP depressed during injection	5 / 5 [#2 #4 #5 #8 #10 #11]	4 / 4.5 [#4 #5 #7 #9]
Pleura lateral to the TP depressed during injection	1 / 3 [#6]	2 / 4 [#10 #11]

[# (Number)] indicates the consecutive number of the cadaver (refer to Fig. 2B)

Data are shown as median

## Discussion

The primary aim of this cadaveric study was to investigate factors influencing injectate spread during ultrasound-guided TPVB, beyond the well-described differences in TPVB approaches [9, 13]. We used an intercostal approach in the lateral decubitus position, which is widely adopted in clinical practice and extensively evaluated in previous studies [8, 9, 13]. It should be noted that TPVB is typically performed with the block side uppermost due to ease of access, and we do not advocate for administration from the lower side in clinical practice. We intentionally maintained the same lateral position for bilateral injections, including the lower-side injection, as a purely experimental design to eliminate the confounding influence of repositioning when assessing laterality. Furthermore, since all procedures were performed by a single experienced operator, and pleural displacement and dye staining were successfully confirmed upon dissection even on the lower side in all cases, we consider that procedural bias was minimized and the validity of the experiment was ensured.

The principal finding of this study was that dye spread was greater on the left side, regardless of whether the injection was performed on the upper or lower side in the lateral decubitus position. Although we cannot rule out the possibility that the study was underpowered to detect subtle positional effects due to the small sample size, we did not observe any consistent trend between the upper and lower sides in this dataset.

This asymmetrical injectate spread may be related to anatomical differences in the TPVS capacity, as described by Kittredge [14]. A larger capacity on the left side may facilitate an ‘extrapleural compartment (EPC)-dominant’ distribution pattern. We also considered the potential role of the endothoracic fascia (ETF), which divides the TPVS into the ventral EPC and the dorsal subendothoracic compartment (SETC) [15]. Injection into the EPC is suggested to promote longitudinal spread, whereas SETC deposition tends to limit it [16]. Although the ETF is macroscopically indistinct [17], the prominent medial pleural displacement observed more frequently on the left side suggests an EPC-dominant distribution, potentially explaining the wider longitudinal spread.

In addition, a previous study demonstrated that the TPVS, extrapleural space, and intercostal nerve plane form a continuous network [18], suggesting that extensive spread within the TPVS may also contribute to injectate diffusion across multiple ICSs.

**Fig. 5:**
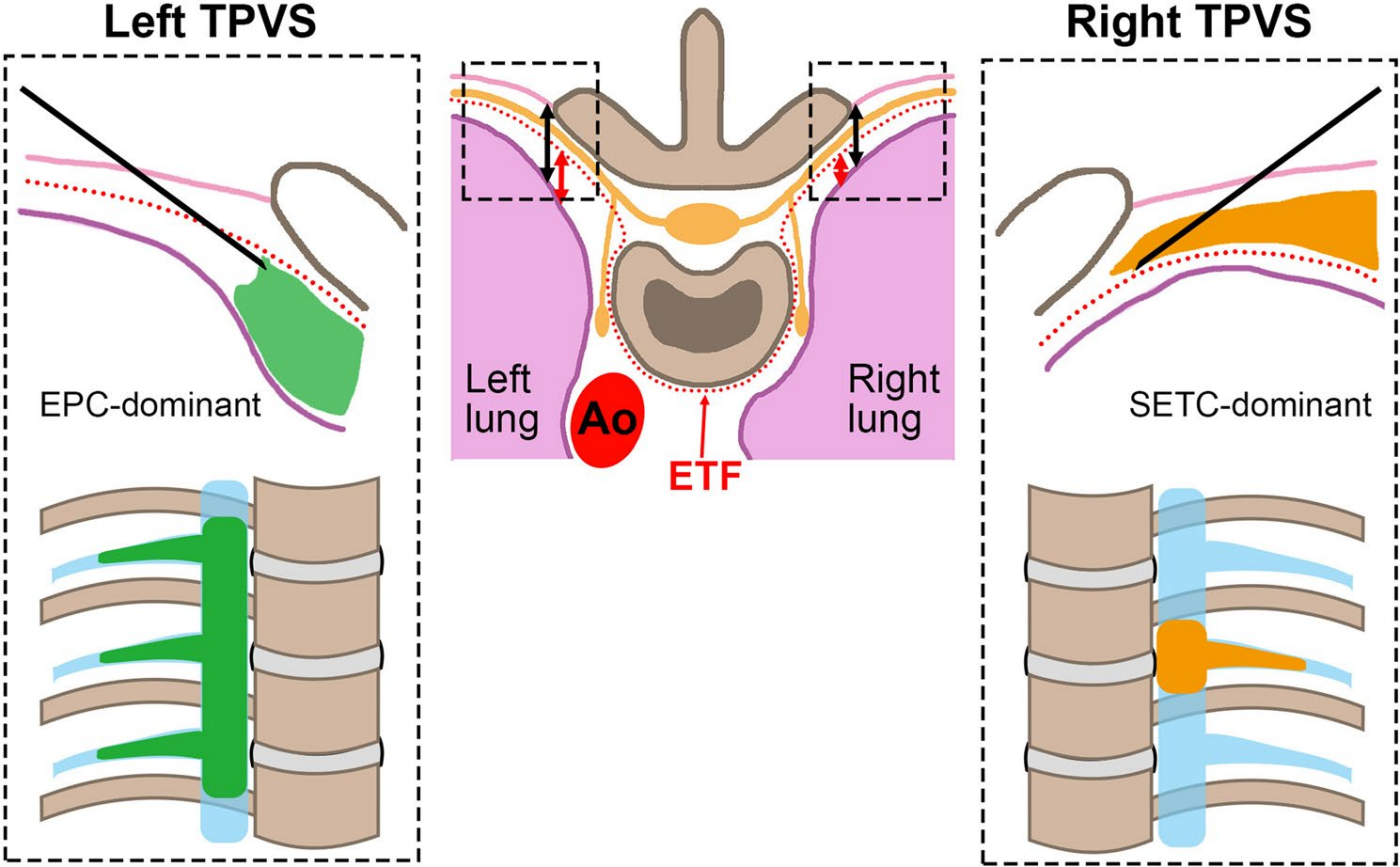
Hypothetical schematic illustrating a possible mechanism underlying left–right asymmetry of the TPVS and differences in injectate distribution patterns. This schematic represents a conceptual model based on the pleural displacement patterns observed in this study. The ETF (red dotted line) is depicted to explain the functional differences in injectate spread patterns between the EPC and the SETC. Differences in the width of the EPC (indicated by double-headed red arrows) suggest a possible explanation for the variation in injectate distribution patterns. Double-headed black arrows indicate the width of the TPVS. Light blue regions represent TPVSs. Green and orange regions represent the injectate administered into the TPVS. Ao: Descending aorta; ETF: Endothoracic fascia; EPC: Extrapleural compartment; SETC: Subendothoracic compartment

Figure 5 illustrates our hypothesized mechanism underlying the laterality of injectate spread. Although speculative in the absence of histological confirmation, this model provides a consistent explanation for the functional differences observed in our study.

The observed asymmetry has potential clinical implications. To achieve extensive spread in right TPVB, it may be more important than in left TPVB to use a multiple-injection technique [7, 19]—which has been demonstrated to provide a wide analgesic range. Another practical option is to confirm an EPC-dominant pattern by observing medial pleural displacement during injection. These considerations may help refine the current understanding of TPVB variability.

Our findings differ from those of Marhofer et al. [8], who reported no significant left–right difference in contrast spread using magnetic resonance imaging. This discrepancy may be related to fundamental differences in tissue mechanics between living subjects and cadavers [7, 20]. In living subjects, physiological factors such as intrathoracic pressure fluctuations and muscle tone may influence the TPVS compliance, potentially masking subtle anatomical asymmetries. Differences in physicochemical properties (e.g., viscosity and pH) between the acrylic dye used in our study and the local anesthetic solution used in the volunteer study could have affected the spread characteristics [17, 20]. In addition, methodological differences could have contributed, as the previous study did not document the precise needle tip location or the pleural displacement patterns.

This study has several limitations. First, the sample size was small; therefore, the study may have lacked sufficient statistical power to detect subtle difference due to positioning. Second, injection levels could not be standardized due to visualization difficulties in some cadavers. Third, we evaluated only an intercostal approach; other approaches might result in different spread patterns. Fourth, the tissue properties of Thiel-embalmed cadavers differ from those of living tissue, and the physicochemical properties of the acrylic dye differ from those of clinical local anesthetics; both factors could potentially affect injectate dynamics. Finally, we did not assess spread into the epidural space or erector spinae muscles, which could also contribute to the overall distribution profile.

## Conclusions

This study demonstrates a significant laterality in injectate spread during TPVB, with a more longitudinal distribution observed on the left side than on the right. Notably, this tendency was evident regardless of whether the injection was performed on the upper or lower side in the lateral decubitus position. Clinically, these findings suggest that employing a multiple-injection technique or strategies to ensure adequate displacement of the medial pleura may be more important in right TPVB than in left TPVB to achieve extensive injectate spread.

Although the precise mechanisms underlying this laterality remain to be directly confirmed, our findings may provide important insights for understanding the previously noted issue of variability in injectate distribution in TPVB. Further investigations from different perspectives, including histological or clinical studies, are warranted to verify this hypothesis.

## Data Availability

The datasets used during the current study are available from the corresponding author upon reasonable request.

## Abbreviations

TPVB: Thoracic paravertebral block
TPVS: Thoracic paravertebral space
TP: Transverse process
ICS: Intercostal space
IIM: Internal intercostal membrane
ETF: Endothoracic fascia
EPC: Extrapleural compartment
SETC: Subendothoracic compartment

## References

[1] Eason MJ, Wyatt R. Paravertebral thoracic block — a reappraisal. Anaesthesia. 1979;34:638–42. 517716 10.1111/j.1365-2044.1979.tb06363.x

[2] Krediet AC, Moayeri N, van Geffen GJ, Bruhn J, Renes S, Bigeleisen PE, et al. Different approaches to ultrasound-guided thoracic paravertebral block: an illustrated review. Anesthesiology. 2015;123:459–74. 26083767 10.1097/ALN.0000000000000747

[3] Lönnqvist PA, MacKenzie J, Soni AK, Conacher ID. Paravertebral blockade. Failure rate and complications. Anaesthesia. 1995;50:813–5. 7573876 10.1111/j.1365-2044.1995.tb06148.x

[4] Patnaik R, Chhabra A, Subramaniam R, Arora MK, Goswami D, Srivastava A, et al. Comparison of paravertebral block by anatomic landmark technique to ultrasound-guided paravertebral block for breast surgery anesthesia: a randomized controlled trial. Reg Anesth Pain Med. 2018;43:385–90. 29462058 10.1097/AAP.0000000000000746

[5] Davies RG, Myles PS, Graham JM. A comparison of the analgesic efficacy and side-effects of paravertebral vs epidural Blockade for thoracotomy — a systematic review and meta-analysis of randomized trials. Br J Anaesth. 2006;96:418–26. 16476698 10.1093/bja/ael020

[6] Ding X, Jin S, Niu X, Ren H, Fu S, Li Q. A comparison of the analgesia efficacy and side effects of paravertebral compared with epidural Blockade for thoracotomy: an updated meta-analysis. PLoS ONE. 2014;9:e96233. 24797238 10.1371/journal.pone.0096233 PMC4010440

[7] Cowie B, McGlade D, Ivanusic J, Barrington MJ. Ultrasound-guided thoracic paravertebral blockade: a cadaveric study. Anesth Analg. 2010;110:1735–9. 20435949 10.1213/ANE.0b013e3181dd58b0

[8] Marhofer D, Marhofer P, Kettner SC, Fleischmann E, Prayer D, Schernthaner M, et al. Magnetic resonance imaging analysis of the spread of local anesthetic solution after ultrasound-guided lateral thoracic paravertebral blockade: A volunteer study. Anesthesiology. 2013;118:1106–12. 23442752 10.1097/ALN.0b013e318289465f

[9] Termpornlert S, Sakura S, Aoyama Y, Wittayapairoj A, Kishimoto K, Saito Y. Distribution of injectate administered through a catheter inserted by three different approaches to ultrasound-guided thoracic paravertebral block: a prospective observational study. Reg Anesth Pain Med. 2020;45:866–71. 32848087 10.1136/rapm-2020-101545

[10] Hara K, Sakura S, Nomura T, Saito Y. Ultrasound guided thoracic paravertebral block in breast surgery. Anaesthesia. 2009;64:223–5. 19143711 10.1111/j.1365-2044.2008.05843.x

[11] Shibata Y, Nishiwaki K. Ultrasound-guided intercostal approach to thoracic paravertebral block. Anesth Analg. 2009;109:996–7. 19690285 10.1213/ane.0b013e3181af7e7b

[12] Taketa Y, Fujitani T. A novel paralaminar in-plane approach for ultrasound-guided continuous thoracic paravertebral block using microconvex array transducer. Reg Anesth Pain Med. 2015;40:390. 26079356 10.1097/AAP.0000000000000259

[13] Taketa Y, Irisawa Y, Fujitani T. Comparison of analgesic efficacy between two approaches of paravertebral block for thoracotomy: a randomized trial. Acta Anaesthesiol Scand. 2018;62:1274–9. 30047132 10.1111/aas.13216

[14] Kittredge RD. Computed tomographic evaluation of the thoracic prevertebral and paravertebral spaces. J Comput Tomogr. 1983;7:239–50. 6884060 10.1016/0149-936x(83)90087-5

[15] Karmakar MK, Chung DC. Variability of a thoracic paravertebral block: are we ignoring the endothoracic fascia? Reg Anesth Pain Med. 2000;25:325–7. 10834797 10.1016/s1098-7339(00)90028-2

[16] Naja MZ, Ziade MF, El Rajab M, El Tayara K, Lönnqvist PA. Varying anatomical injection points within the thoracic paravertebral space: effect on spread of solution and nerve Blockade. Anaesthesia. 2004;59:459–63. 15096240 10.1111/j.1365-2044.2004.03705.x

[17] Bouman EAC, Sieben JM, Balthasar AJR, Joosten EA, Gramke HF, Van Kleef M, et al. Boundaries of the thoracic paravertebral space: potential risks and benefits of the thoracic paravertebral block from an anatomical perspective. Surg Radiol Anat. 2017;39:1117–25. 28444433 10.1007/s00276-017-1857-4 PMC5610675

[18] Ohgoshi Y, Usui Y, Terada S, Takeda Y, Ohtsuka A, Matsuno K, et al. Visualization of injectate spread of intercostal nerve: a cadaveric study. JA Clin Rep. 2018;4:65. 32026062 10.1186/s40981-018-0204-z PMC6967250

[19] Naja ZM, El-Rajab M, Al-Tannir MA, Ziade FM, Tayara K, Younes F, et al. Thoracic paravertebral block: influence of the number of injections. Reg Anesth Pain Med. 2006;31:196–201. 16701182 10.1016/j.rapm.2005.12.004

[20] Behr AU, Chan VWS, Stecco C. Living versus cadaver fascial plane injection. Reg Anesth Pain Med. 2020;45:156–7. 10.1136/rapm-2019-100893 31511366

